# Inhibition of Connexin 43 Hemichannels Alleviates Cerebral Ischemia/Reperfusion Injury via the TLR4 Signaling Pathway

**DOI:** 10.3389/fncel.2018.00372

**Published:** 2018-10-17

**Authors:** Yingzhu Chen, Liangzhu Wang, Lingling Zhang, Beilei Chen, Liu Yang, Xiaobo Li, Yuping Li, Hailong Yu

**Affiliations:** ^1^Clinical Medical College of Yangzhou University, Yangzhou, China; ^2^Department of Neurology, Northern Jiangsu People’s Hospital, Yangzhou, China; ^3^Dalian Medical University, Dalian, China; ^4^Institute of Neuroscience, Northern Jiangsu People’s Hospital, Yangzhou, China; ^5^Drum Tower Hospital, Medical School of Nanjing University, Nanjing, China

**Keywords:** cerebral ischemia/reperfusion, hemichannel, inflammation, toll-like receptor 4, neurovascular unit, neuroprotection

## Abstract

Connexin 43 (Cx43) widely exists in all components of the neurovascular unit (NVU) and is a constituent of gap junctions and hemichannels. In physiological states, gap junctions are open for regular intercellular communication, and the hemichannels present low open probability in astrocytes. After cerebral ischemia, a large number of hemichannels are unusually opened, leading to cell swelling and even death. Most known hemichannel blockers also inhibit gap junctions and sequentially obstruct normal electrical cell-cell communication. In this study, we tested the hypothesis that Gap19, a selective Cx43-hemichannel inhibitor, exhibited neuroprotective effects on cerebral ischemia/reperfusion (I/R). An obvious improvement in neurological scores and infarct volume reduction were observed in Gap19-treated mice after brain ischemia induced by middle cerebral artery occlusion (MCAO). Gap19 treatment attenuated white matter damage. Moreover, Gap19 treatment suppressed the expression of Cx43 and Toll-like receptor 4 (TLR4) pathway-relevant proteins and prevented the overexpression of tumour necrosis factor-α (TNF-α) and interleukin-1β (IL-1β). To further explore downstream signaling, we established an *in vitro* model–oxygen glucose deprivation (OGD) to simulate ischemic conditions. Immunofluorescence staining showed that Cx43 co-existed with TLR4 in astrocytes. The hemichannel activity was increased after OGD and Gap19 could inhibit this effect on astrocytes. Gap19 substantially improved relative cell vitality and decreased the expression of Cx43, TLR4 and inflammatory cytokines *in vitro*. In addition, in the lipopolysaccharide (LPS) stimulation OGD model, Gap19 also exhibited a protective effect via inhibiting TLR4 pathway activation. In summary, our results showed that Gap19 exerted a neuroprotective effect after stroke via inhibition of the TLR4-mediated signaling pathway.

## Introduction

Stroke is the most common neurological disease, and it causes high disability and high mortality (Lo, 2010). An epidemiological study revealed that 70%–80% of all strokes are ischemic (Mozaffarian et al., 2015). Currently, intravenous tissue plasminogen activator (t-PA) is recognized as the most effective therapy for acute ischemic stroke (Powers et al., 2018). However, its clinical use is largely limited because of the narrow time window and strict inclusion criteria (Saver et al., 2013). Recent research has been devoted to the development of alternative neuroprotective agents, but almost no agents have been successful in clinical trials (Sahota and Savitz, 2011). The primary reason is that previous studies focus on single target neurons or white matter and ignore the communication between the components of the neurovascular unit (NVU; Girouard and Iadecola, 2006). NVU is a complex of neurons, astrocytes, and cerebrovascular endothelial cells, considered as the structural and functional unit of the brain (Zhang et al., 2012; Xue et al., 2013). NVU components are involved in the transport of substances via intercellular signal transduction and neurovascular coupling and regulate the steady state microenvironment of neurons (Guo et al., 2008). Abnormal NVU function may cause neurological disorders, such as stroke (Moskowitz et al., 2010).

Cell communication in the NVU primarily occurs via gap junctions (Islam and Mohamed, 2015). Gap junctional intercellular communication (GJIC) allows direct cell-to-cell communication, energy metabolites and diffusion of molecules to maintain homeostatic balance in the brain, such as K^+^ or glutamate (Naus and Giaume, 2016). Gap junction is consisted by the aggregation of two connexons (or hemichannels), to form a direct pathway linking the cytoplasm of the neighboring cells (Bodendiek and Raman, 2010). Each hemichannel consists of six connexin proteins, and 11 subtypes are expressed in brain (Chew et al., 2010). The connexin 43 (Cx43) subtype is one of the most abundant connexin proteins in brain, widely exists in the component of NVU, predominately in astrocytes (Giaume et al., 2010). Connexins are four-time transmembrane proteins and exist on the plasma membranes. They own two extracellular loops (ELs) and one intracellular cytoplasmic loop (CL; Schulz et al., 2015). In the physiological state, gap junctions in astrocytes remain open, while the hemichannels still present low open probability (Kim et al., 2016). Hemichannels are primarily activated after cerebral ischemia, allowing the entry of Na^+^ and Ca^2+^ and the release of adenosine triphosphate (ATP) and other small metabolites. These fluctuations cause Ca^2+^ overload, osmotic imbalance, energy exhaustion and even cell death (Sáez and Leybaert, 2014). Moreover, ATP released from hemichannels could mediate the microglia activation and induce inflammatory cytokines secretion from activated microglia, further upregulate the Cx43 hemichannels and form a vicious cycle (Shaikh et al., 2012; Sáez et al., 2013).

Hemichannels modulation was considered as a potential neuro-therapeutic target in cerebral ischemia/reperfusion (I/R) injury (De Bock et al., 2011). Connexin43 mimetic peptides could prevent the opening of hemichannels and further reduce the spread of harmful substance after ischemic injury (Evans and Leybaert, 2007; O’Carroll et al., 2008). Our previous study found that the Gap26 and Gap27 could significantly reduce infarct size and promote neurological function recovery after hypoxia/ischemia injury in neonatal rats (Li et al., 2015). However, when applied for several hours, these agents also prevented the joining of hemichannels and affected gap junction communication due to their poor specificity (Decrock et al., 2009). Another mimetic peptide, Gap19, consists of nine amino acids KQIEIKKFK (Schulz et al., 2015), and it inhibits the opening of hemichannels without interference the gap junction communication in astrocytes (Abudara et al., 2014). Selective inhibition of Cx43 hemichannels using Gap19 protects against myocardial I/R injury *in vitro* and *in vivo* (Wang N. et al., 2013). Given the higher specificity, we can hypothesis that Gap19 may alleviate cerebral I/R injury. Restoration of blood supply and reoxygenation are usually associated with deterioration of tissue injury and a profound inflammation called reperfusion injury (Kim et al., 2014). Subsequent reperfusion further activates of innate and adaptive immune responses and cell death programmes (Eltzschig and Eckle, 2011). Recently study shows that Cx43 hemichannel opening could be triggered by treatment with pro-inflammatory cytokines in astrocytes (Retamal et al., 2007). Nevertheless, whether Gap19 influences cerebral I/R injury via disruption of inflammatory responses is not clear.

In the present study, we established middle cerebral artery occlusion (MCAO) and oxygen glucose deprivation (OGD) models to assess the role and relevant mechanisms of Gap19 in cerebral ischemia. We found that Gap19 exerted a protective role in cerebral I/R injury via inhibition of Toll-like receptor 4 (TLR4) signaling *in vitro* and *in vivo*. Therefore, Gap19 is a promising new drug candidate for the treatment of ischemic stroke.

## Materials and Methods

### Animals and Experimental Groups

Adult male ICR mice (8–10 weeks, 25–35 g) were purchased from the Comparative Medical Centre of Yangzhou University. Animals were housed in an appropriate environment at 22 ± 2°C and 60% humidity with a 12-h light/dark cycle. All experiment protocols were approved by the Animal Ethics Committee of the Yangzhou University (license number: YIACUC-15-0013). Animals were randomly separated into five groups (*n* = 6–15 each): (I) sham; (II) Gap19 group; (III) I/R group; (IV) I/R + Gap19 group; and (V) I/R + Gap26 group. In the I/R group, the animals were assigned to several subgroups with different reperfusion time points (4 h, 12 h, 24 h, 72 h and 7 days).

### The Middle Cerebral Artery Occlusion (MCAO) Model

The MCAO was established as described previously (Wang et al., 2014). Briefly, after anesthetize mice with 5.0% isoflurane, made a small incision on the neck skin, and carefully exposed the left common carotid artery (CCA), external carotid artery (ECA), and internal carotid artery (ICA). The ECA was ligated to block blood flow, and aneurysm clips were used to clamp the left CCA and ICA. A small incision was created at 2 mm proximal end of the CCA bifurcation. Through the incision, the filament was inserted into the ICA, and about 9–11 mm was inserted to block the MCA. The filament was gently removed (onset of reperfusion) after 45 min of focal cerebral ischemia. Tight the ICA with suture line and close the neck incision. Sham group only accepted neck incision and ligation of the ECA without occluding MCA. The mice went back to the cages with closely monitored and keep the body temperature at 36.5–37.5°C by the electric blanket until they recovered from anesthesia.

### Drug Preparation and Treatment Schedule

Gap19 was purchased from TOCRIS (catalog No. 5353) and dissolved in sterilized double distilled water (ddH_2_O). Gap19 was injected into the right lateral cerebral ventricle (coordinates: 0.5 mm posterior to Bregma, 1.0 mm right of the midline, and depth is 2.5 mm) 1 h after MCAO as described previously (Meller et al., 2005). The optimal dose was selected based on previous studies (Li et al., 2015) and 10 μg Gap19/Gap26 in 10 μl ddH_2_O was slowly injected over 10 min.

### Evaluation of Neurological Deficit

A researcher who was unclear of animal grouping assessed neurological deficits at 24 h after reperfusion. A 5-point method of neurological deficit scores was applied to assess neurological behavior, as described previously (Yu H. et al., 2012): 0, no deficit; 1, failure to fully extend right paw; 2, circling to right; 3, falling to right; 4, no spontaneous walking with depressed consciousness. Score of 0–2 manifested mild neurological impairment, and 3–4 manifested severe neurological impairment. Remove the animals with no deficit after MCAO.

### Measurement of Infarct Volume

Infarct volume was measured with 0.2% (w/v) 2,3,5-triphenyltetrazolium chloride (TTC, Sigma-Aldrich) as described previously (Tsubokawa et al., 2007; Yu H. et al., 2012). Briefly, mice were decapitated after neurological evaluation, and quickly removed and frozen the brain. After cut the brains into 2 mm coronal sections, slices were stained with 0.2% TTC for 30 min at 37°C, then fixed with 4% paraformaldehyde overnight. The infarct tissue area was not stained (white), and normal tissue was stained red. Then all brain slices were photographed in one picture and the infarct volume in each slice was determined by a computerized image analysis system (AlphaEase Image Analysis Software V 3.1.2). The percent hemispheric infarct volume was calculated as described previously (Xiong et al., 2011; Li et al., 2015), calculated the ratio (%) of the infarction area to the contralateral area of the same brain.

### Luxol Fast Blue Myelin Staining

Luxol fast blue staining (Sigma) and Cresyl violet was used as a counterstain to assess the myelin damage at 7 days after reperfusion. Washed the slices in ddH_2_O for 2 min, and incubated in 95% ethanol for 1 min. Sections were then incubated in a preheated solution of 1% LFB (60°C) for 2 h. After washed, differentiate sections with a 0.05% lithium carbonate solution and 70% ethyl alcohol each for 10 s. Repeated this step twice and stained with a 0.1% cresyl violet for 1 min. Tissues were gradually dehydrated in an ethanol gradient (70%, 95% and 100%) and fixed with xylene. Quantitative analyses of Luxol fast blue-stained sections were performed as described previously (Jing et al., 2014). White matter damage was calculated as the percentage of blue area in the ipsilateral striatum.

### Isolation and Purification of Three Kinds of Mice Cerebral Cells

Brain microvascular endothelial cells (BMECs) were collected from 2-week-old mice, as previously described (Gauthier et al., 2012; Ruck et al., 2014). Briefly, gray matter was minced into small pieces in ice-cold 20 mM HEPES in Dulbecco’s Modified Eagle Medium (DMEM). Tissue was digested at 37°C for 30 min in 0.05% collagenase/dispase. Isolated BMECs with 17% dextran and ultracentrifuged at 4°C 10,000× *g* for 30 min. Autoclaved glass beads were used to collect the cells. Precipitates were resuspended in DMEM supplemented with 10% FBS, 100 U/ml penicillin, 100 mg/ml streptomycin, 2 mM L-glutamine, 50 mg/ml DNase I and 0.1 mM nonessential amino-acids. Seed the cells on 6-well culture plates pre-coated with 1% gelatin and incubate at 37°C in 5% CO_2_. The purified endothelial cells were used for following experiments.

Astrocytes were prepared from mice brain cortices within 24 h of birth, as previously described (Nakagawa et al., 2009). Gray cortices were minced, digested 0.25% trypsin and centrifuged. Sediments were resuspended in DMEM supplemented with 10% FBS and 100 U/ml penicillin, and 100 mg/ml streptomycin. Cells were seeded at a density of 4.5 × 10^6^ cells/flask onto 75-cm^2^ flasks pre-coated with 20 μg/ml poly-D-lysine. Change the culture medium every 2 days. After 12–14 days of culture, shake the flasks to discard microglia and collect the purified astrocytes. Astrocytes could be digested with 0.25% trypsin and seeded on different culture plates for further experiments.

Neurons were obtained from mice embryos at 15–16 days of gestation, as previously described, using a modified protocol (Zhu et al., 2014). Briefly, remove the embryos and separate the cerebral cortex quickly, strip meninges in the sterile filter papers. Put the acquired tissues into a Ca^2+^/Mg^2+^-free Hank’s balanced saline solution (HBSS) solution. Then digest in 2-mg/ml papain for 30 min and centrifuged. Cells were seeded at 5 × 10^5^ cells/cm^2^ into 6-well culture plates pre-coated with 100 μg/ml poly-D-lysine and cultured with Neurobasal medium including 4.5 g/L glucose, Glutamax and 2% B27. Replace half of the medium with new medium after 2 days and maintain in culture 3 days prior to each experiment.

### Establishment of *in vitro* NVU Model

NVU model was constructed by using the transwell system (Corning Incorporated) according to previous report (Xue et al., 2013; Tian et al., 2016). Briefly, the neurons were first seeded into the 6-well culture plate to 0.5 × 10^5^ cells/cm^2^ for 5 days. Then seed the purified astrocytes on the external of the transwell insert membrane with a density of 5 × 10^5^ cells/cm^2^. After astrocytes were adhered for 4 h, and the insert was transferred into the well to co-culture with neurons. After 1 day, BMECs were plated to the internal of the transwell insert membrane with 1.0 × 10^5^ cells/cm^2^ and co-cultured for 2–3 days. After co-culture, medium was replaced with DMEM-F12 supplemented with 10% FBS, L-glutamine, penicillin (100 U/ml) and streptomycin (100 mg/ml). The NVU model was prepared for next experiments. Lipopolysaccharide (LPS; 1 μg/ml, Sigma) and Gap19 were added to the medium below the transwell at the beginning of OGD to construct the model.

### Oxygen-Glucose Deprivation

To simulate ischemia *in vitro*, OGD treatment was established as described previously (Rowe et al., 2014) with small modification. First, removed the original culture medium and replaced with glucose/serum-free DMEM. Then, transfer the plates into an anaerobic chamber for 4 h at 37°C, which already balanced with 5% CO_2_ and 95% N_2_. Cells were returned to completely normal conditions for 24 h. Groups as follows: (1) control; (2) Gap19 group; (3) OGD group; (4) OGD + Gap19 group; (5) OGD + LPS group; and (6) OGD + LPS + Gap19 group. Control cells were incubated in the normoxic incubator. Gap19 (100 μM) were treated into system at the beginning of OGD.

### Cytotoxicity Assay

The 3-(4,5-dimethyl-thiazol-2-yl)-2,5-diphenyltetrazolium bromide (MTT) assay was used to assess the cell viability (Jiang et al., 2014). Briefly, purified astrocytes were seeded in 96-well plates with DMEM without serum. Cells were disposed with different concentrations (10 μM, 50 μM, 100 μM, 150 μM and 200 μM) of Gap19 at the beginning of OGD. Then the medium was removed, and the MTT assay was conducted according to the manufacturer’s protocols. The formation of formazan crystals was detected at 490 nm by a microplate reader. Cell viability was showed as a percentage of the control that had not undergone Gap19 treatment.

### Immunofluorescence Staining

Immunofluorescence was performed as described in a previous study (Tian et al., 2016). Briefly, different cell types were seeded on coverslips. Cells were gently washed after reaching 70% confluency and fixed with 4% paraformaldehyde for 20 min. Then block with 3% bovine serum for 1 h and incubate with primary antibody at 4°C overnight. BMECs were incubated with CD31 (anti-rabbit, 1:20, Abcam), and neurons were incubated with tubulin β-III isoform (anti-mouse, 1:200, Millipore). Astrocytes were incubated with GFAP (anti-mouse, 1:100, Millipore; anti-rabbit, 1:500, Cell Signaling), Cx43 (anti-rabbit, 1:1,000, Cell Signaling; anti-mouse, 1:100, Abcam) and TLR4 (anti-mouse, 1:500, Proteintech). Cells were washed and incubated with anti-rabbit IgG labeled with Alexa-594 (Invitrogen) or anti-mouse IgG labeled with Alexa-488 (Invitrogen). Finally, stain the nuclei with 4’,6-diamidino-2- phenylindole (DAPI, C1006; Beyotime, Shanghai, China). The coverslips were viewed by the fluorescence microscope (LSM780, Zeiss, Jena, Germany).

### Ethidium Bromide Uptake

To evaluate the hemichannels activity after OGD and the effect of Gap19, astrocytes, neurons and BMECs were respectively cultured on 24-well plates. After OGD, the cultured cells were washed with HBSS twice and incubated with 5 μM ethidium bromide (EtBr; Giaume et al., 2012) in 37°C for10 min. Then washed the cells again, fixed with 4% paraformaldehyde and visualized by the fluorescence microscope. Pictures were analyzed through counting the number of EtBr-positive cells per field by ImageJ software.

### Enzyme-Linked Immunosorbent Assay (ELISA) Analysis

Carefully collect the cell culture supernatants after OGD and centrifuge at 12,000× *g* 4°C for 15 min. Tumor necrosis factor-α (TNF-α) and interleukin-1β (IL-1β) concentrations were evaluated by Enzyme-Linked Immunosorbent Assay (ELISA) assay kits. All procedures were performed following the directions provided in the kit. The absorbance of each sample was detected by a microplate reader at 490 nm. The concentration of cell cytokines is described as picograms per milliliter.

### Western Blot Analysis

At different reperfusion times, protein was extracted from the infarcted hemispheres and cultured cells by a protein extraction kit (Beyotime Biotech) as described in our previous report (Li et al., 2015). Protein samples were next electrophoresed and transferred to polyvinylidene difluoride filter (PVDF) membranes. Then block the membrane with 5% fat-free milk at room temperature for 2 h, and incubate with primary antibodies as follows: anti-Tubulin (Cell Signaling), anti-Cx43 (Cell Signaling), anti-TLR4 (Proteintech), anti-MyD88 (Cell Signaling), anti-NF-κB p65 (Proteintech), anti-IL-1β (Bioss), and anti-TNF-α (Bioss) at 4°C overnight. Next day, incubate with secondary horse anti-mouse or goat anti-rabbit antibodies conjugated with horseradish peroxidase (Cell Signaling) for 2 h and visualize by an enhanced chemiluminescence system (ECL). Relative protein levels were quantified after normalization to Tubulin.

### Statistical Analysis

All data were described as the mean ± SEM. Pictures were analyzed by ImageJ software. Data from all experiments were quantified and analyzed by GraphPad Prism 7.0 software. *P* < 0.05 was regarded as statistically significant.

## Results

### Gap19 Decreased Infarct Volume and Prevented the Deterioration of Neurological Deficit

**Figure 1:**
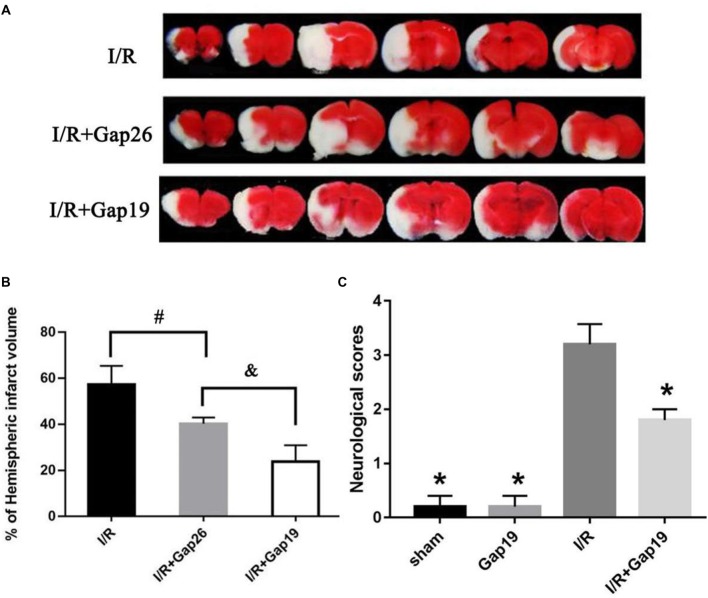
Gap19 decreased infarct volume and prevented the deterioration neurological deficit after ischemia/reperfusion (I/R) injury. Mice were administered Gap26 or Gap19 1 h after middle cerebral artery occlusion (MCAO). 2,3,5-triphenyltetrazolium chloride chloride (TTC) staining and neurological scores were examined at 24 h after reperfusion. **(A)** Representative TTC-stained slices of different treatment groups after MCAO. The infarct tissue area was not stained (white), and normal tissue was stained (red). **(B,C)** Statistical analyses of infarct volume of different treatment groups were shown. **(C)** Neurological scores were assessed with a 5-point scale system. Gap19 treatment observably decreased neurological deficits. Data represent mean ± SEM of five brains. *P < 0.05 vs. I/R group, ^#,&^*P* < 0.05.

Animals were euthanized after 24 h reperfusion, and the brain infarction volume was analyzed using TTC staining to determine whether Gap19 was protective after cerebral I/R injury. Results demonstrated I/R group has a large infarct volume (56.06 ± 1.77%). Gap26 and Gap19 treatment groups exhibited a significantly smaller infarct volume than I/R group (*P* < 0.05, Figures 1A,B). Furthermore, the infarct volume of the I/R + Gap19 group (27.9 ± 0.93%) was significantly smaller than that of the I/R + Gap26 group (40.12 ± 0.95%; *P* < 0.05, Figures 1A,B). This result demonstrated that Gap19 had a better neuroprotective effect. As shown in Figure 1C, no obvious neurological deficits were generated in the sham or Gap19 groups, whereas severe deficits could be observed in I/R group. Consistent with this result, we also found that Gap19 treatment remarkably reduced neurological scores and prevented the deterioration of the neurological function (*P* < 0.05, Figure 1C).

### Gap19 Ameliorated White Matter Injury After Cerebral I/R Injury

**Figure 2:**
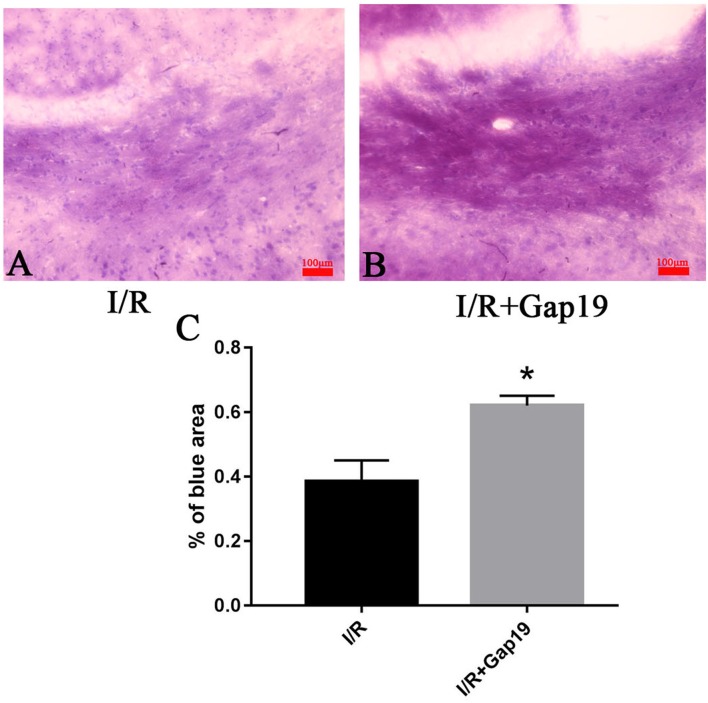
Gap19 ameliorated white matter injury.** (A,B)** Representative pictures of LFB stain at 7 days after cerebral ischemia. **(C)** The percentage of blue area in I/R + Gap19 group was increased compared to I/R group. *P < 0.05 vs. I/R group. Data represent the mean ± SEM (*n* = 3 mice/group). Scar bar = 100 μm.

We euthanized mice at 7 days after reperfusion, and the brains were prepared for frozen sectioning, stained with LFB to examine the influence of Gap19 on white matter after I/R injury. We chose the white matter-enriched area of the striatum for LFB stain and analyzed the blue area in the striatum. Striatum staining was shallow in the I/R group, and the cells were swollen and disorganized (Figures 2A,B). The blue area in I/R + Gap19 group was obviously larger than the area in I/R group (*P* < 0.05, Figure 2C). This result demonstrated that Gap19 alleviated the white matter injury in the MCAO model.

### The Relative Amount of Cx43 and TLR4 Increased After Cerebral I/R Injury

**Figure 3:**
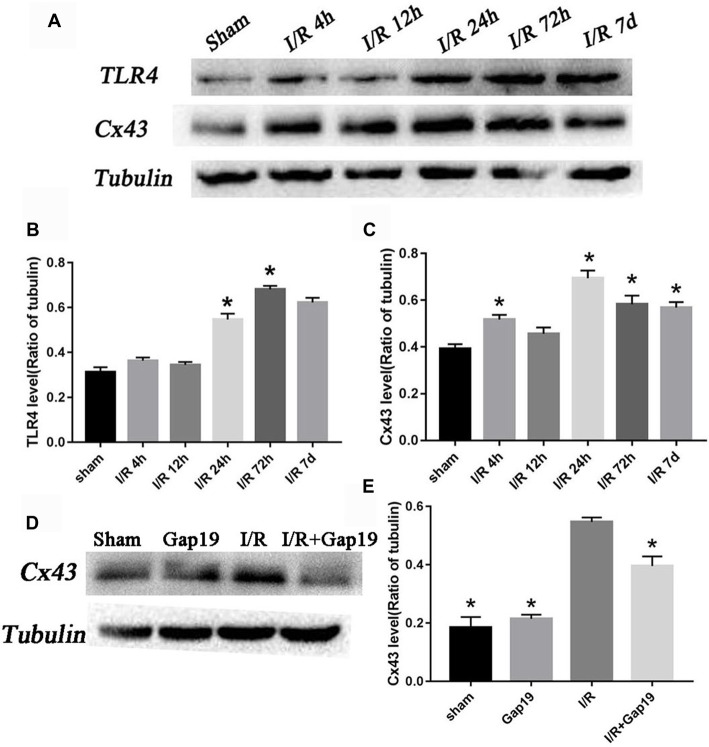
The relative amount of connexin 43 (Cx43) and Toll-like receptor 4 (TLR4) increased after cerebral I/R injury. **(A)** Photomicrographs showed Cx43 and TLR4 tubulin levels in the sham group and 4 h, 12 h, 24 h, 72 h and 7 days after cerebral I/R injury. **(B,C)** Quantitative analyses revealed the variation tendency of Cx43 and TLR4 after cerebral I/R injury. **(D)** Photomicrographs showed Cx43 levels at 24 h after cerebral I/R injury. **(E)** Quantitative analysis of Cx43. Data represent the mean ± SEM (*n* = 3 mice/group). *P < 0.05 vs. I/R group.

Western blots analyzed the expression levels of Cx43 and TLR4 at different reperfusion time points. The infarcted hemispheres were extracted from the sham group and groups with different reperfusion times (4 h, 12 h, 24 h, 72 h and 7 days). Upon I/R 4 h, it was observed an increase in the abundance of Cx43. Expression increased 4 h after reperfusion, peaked 24 h after reperfusion, and declined but remained higher than normal until 7 days (*P* < 0.05, Figures 3A,C). TLR4 level increased obviously in the infarcted region at 24 h after reperfusion and reached a maximum at 72 h post-reperfusion (*P* < 0.05, Figures 3A,B). This trend correlated with Cx43 expression. At 24 h after cerebral I/R injury, the level of Cx43 protein in I/R + Gap19 group was lower than that in the I/R group, which indicated that Gap19 effectively prevented the increase in the abundance of Cx43 (*P* < 0.05, Figures 3D,E).

### Gap19 Inhibited Activation of the TLR4 Pathway and Reduced Inflammatory Cytokines *in vivo*

**Figure 4:**
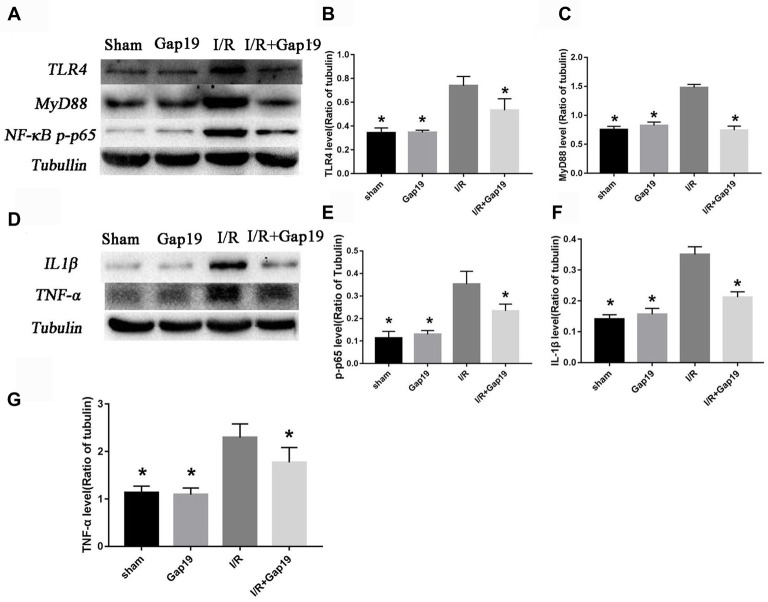
Gap19 suppressed the TLR4 signaling pathway and inflammatory cytokines expression *in vivo*. **(A)** Brain tissues were collected after 24 h reperfusion, and TLR4, MyD88 and NF-κB p-p65 levels were determined by Western blot. Gap19 treatment inhibited the increment of TLR4 **(B)**, MyD88 **(C)** and NF-κB p-p65 **(E)** expression. **(D)** Interleukin-1β (IL-1β) and tumor necrosis factor-α (TNF-α) protein expression was also lower in Gap19-treated mice than I/R mice 24 h post-reperfusion **(F,G)**. Data represent the mean ± SEM (*n* = 3 mice/group). *P < 0.05 vs. I/R group.

The secondary inflammatory response is the main reason for secondary injury after cerebral I/R, and TLR4-mediated innate immunity plays an important role (Wang Y. et al., 2013). We found that the expression of TLR4 was remarkably increased after MCAO than sham group or Gap19 group (*P* < 0.05, Figures 4A,B). TLR4 level was significantly lower in I/R + Gap19 group than I/R group. Gap19 also inhibited the expression of the TLR4 downstream protein MyD88 and NF-κB (*P* < 0.05, Figures 4A,C,E). Furthermore, proinflammatory cytokines as TNF-α and IL-1β released from ischemic tissue are associated with neurotoxic effects by inducing apoptosis of neuronal cells (Boutin et al., 2001; Shichita et al., 2012). Previous studies showed that these cytokines could also affect Cx43 abundance in both astrocytes (Même et al., 2006; Retamal et al., 2007) and microglia (Eugenín et al., 2001). Our study demonstrated that Gap19 treatment remarkably decreased the expression of IL-1β and TNF-α (*P* < 0.05, Figures 4D,F,G), which could further decreasing the Cx43 expression. These results revealed that Gap19 could effectively inhibited TLR4 pathway and decreased inflammation in a mouse cerebral I/R model.

### Cx43 Co-existed With TLR4 in Astrocytes

**Figure 5:**
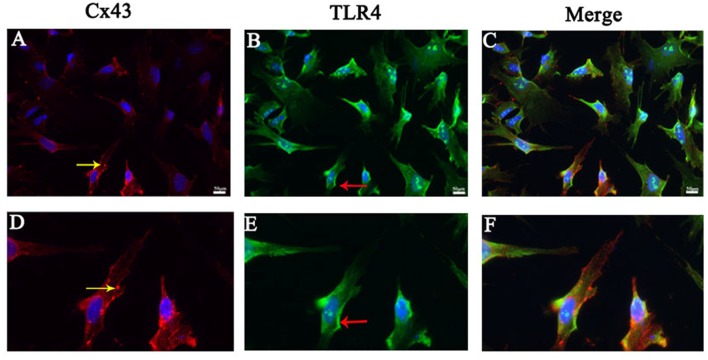
Cx43 co-existed with TLR4 in astrocytes.** (A–C)** Double immunofluorescence staining showed the Cx43 (yellow arrow) and TLR4 (red arrow) in cultured astrocytes after oxygen glucose deprivation (OGD). **(D–F)** The pictures were the amplification of the cell with the arrow above. Scar bar = 50 μm.

We performed immunofluorescence staining for Cx43 and TLR4 in primary astrocytes to further determine the relationship between the protective effect of Gap19 and the TLR4 signaling pathway in cerebral ischemic injury. We could saw both TLR4 and Cx43 existed on astrocytes in the OGD model. Cx43 was primarily expressed in the cytoplasm (yellow arrow), and TLR4 was expressed in the cell membrane (red arrow; Figure 5).

### Gap19 Increased Cell Viability After OGD and Inhibited Cx43 Expression

**Figure 6:**
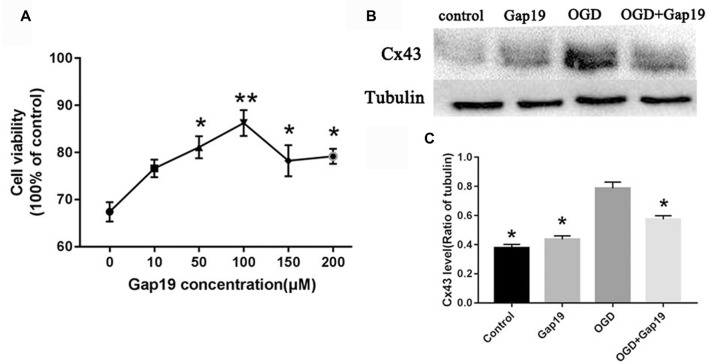
Gap19 increased cell viability after OGD and inhibited Cx43 expression. **(A)** Cell viability following different concentrations of Gap19 after OGD. **(B)** Astrocytes were deprived oxygen-glucose for 4 h following 24 h reoxygenation, and the level of Cx43 was detected using Western blot. **(C)** Quantitative analysis confirmed that Gap19 treatment at the beginning of OGD significantly decreased Cx43 expression. Data represent the mean ± SEM (*n* = 3). *P < 0.05, **P < 0.01 vs. OGD group.

We performed MTT assay in primary astrocytes to determine the optimal concentration of Gap19 in the cell OGD model. Different concentrations of Gap19 were selected to intervention in the onset of OGD, and cell viability was determined. The results demonstrated that Gap19 effectively improved the relative vitality of co-cultured cells at 50 μM, 100 μM, 150 μM and 200 μM (*P* < 0.05, Figure 6A), and we chose optimal concentration—100 μM (*P* < 0.01) for subsequent experiments. The expression level of Cx43 was increased in the cell OGD model, and the expression was reduced after Gap19 intervention, which was consistent with the *in vitro* results (*P* < 0.05, Figures 6B,C).

### Gap19 Decreased the Hemichannels Activity After OGD on Astrocytes

**Figure 7:**
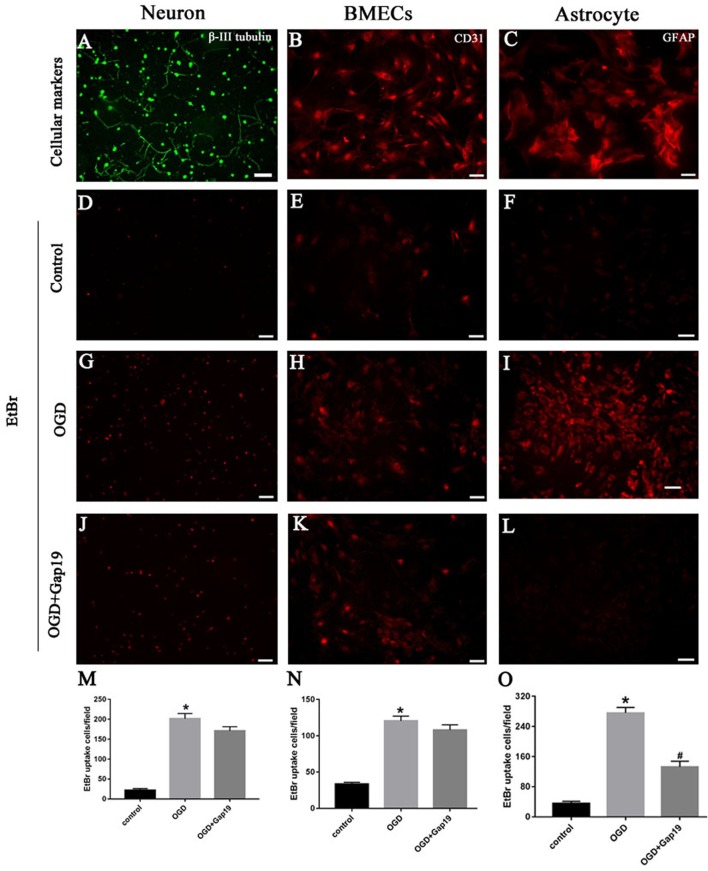
Gap19 decreased the hemichannels activity after OGD on astrocytes. **(A–C)** Immunophenotyping of three cells were identified by β-III tubulin, CD31 and GFAP. **(D–L)** Representative pictures showed ethidium bromide (EtBr) uptake via hemichannels in the three groups of different cells. **(M–O)** EtBr uptake was increased after OGD injury, and Gap19 could remarkably inhibited hemichannels activity. *P < 0.05 vs. control group. ^#^*P* < 0.05 vs. OGD group. Scar bar = 100 μm.

Previous study showed that Gap19 can inhibit Cx43 hemichannel activity in astrocytes after treatment with pro-inflammatory cytokines (Abudara et al., 2014). In this study, we targeted the Cx43 hemichannels after brain ischemia, so we conducted the EtBr uptake assay to evaluate the hemichannels activity in different cells after OGD. First, we identified the three cell types with specific cellular markers. Neuron was verified by anti-β-III tubulin antibody (Figure 7A), BMECs were characterized by anti-CD31 antibody (Figure 7B) and astrocytes were identified by anti-GFAP antibody (Figure 7C). Then, we observed that EtBr uptake was increased in three type cells after OGD (Figures 7G–I) than the control group (Figures 7D–F). There was slightly reduction of hemichannels activity in neurons and BMECs after treatment with Gap19, but without statistical difference (Figures 7J,K,M,N). Meanwhile, Gap19 could obviously inhibit the hemichannels opening after OGD in astrocytes (Figures 7L,O). This result was consistent with a recently study. Walrave et al. (2018) found that Gap19 inhibited the hemichannels open induced by Pilocarpine and exerted anticonvulsant effects. Based on these result, we think astrocytes are the most critical cell type in this triple co-culture system.

### Gap19 Also Prevented the Activation of TLR4 Pathway and Reduced Inflammatory Cytokines *in vitro*

**Figure 8:**
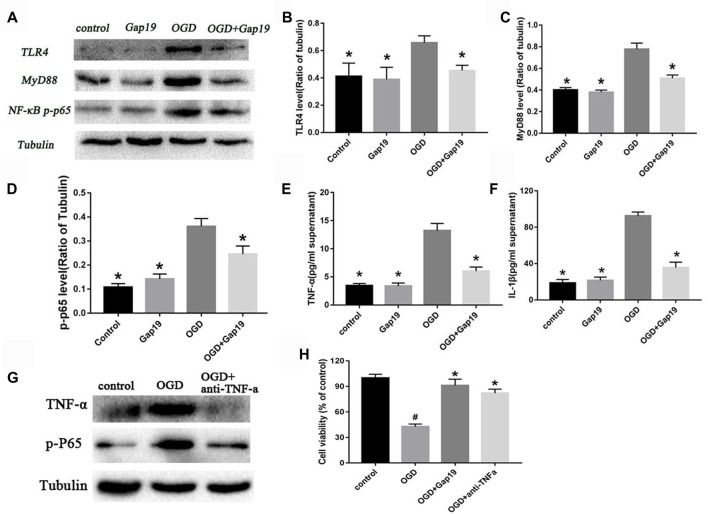
Gap19 prevented the activation of TLR4 pathway and reduced inflammatory cytokines *in vitro*.** (A–D)** Western blot and quantitative analysis showed the expression of TLR4, MyD88 and NF-κB p-p65 were decreased by Gap19 after OGD. **(E,F)** Enzyme-linked immunosorbent assay (ELISA) revealed that Gap19-treated cells exhibited altered release of IL-1β and TNF-α 24 h after reoxygenation. **(G,H)** The neutralizing antibodies of TNF-α decrease the p-P65 expression and increased the astrocytes viability after OGD. Values (mean ± SEM) are representative of at least three independent experiments. *P < 0.05 vs. the OGD group. ^#^*P* < 0.05 vs. the control group.

We constructed a triple cell co-culture system to verify the role of Gap19 *in vitro* cerebral I/R model. Gap19 reduced the activation of TLR4 (*P* < 0.05, Figures 8A,B) compared to the levels in the triple cell co-culture OGD group. MyD88 and NF-κB level were also lower in the OGD + Gap19 group (*P* < 0.05, Figures 8A,C,D). We conducted ELISA to analyze the levels of inflammatory factors in the culture supernatant. The secretion of TNF-α and IL-1β in the Gap19 treatment group was decreased (*P* < 0.05, Figures 8E,F). These results were similar to those in the *in vitro* study. To further identify the anti-inflammation role of Gap19, we use the neutralizing antibodies of TNF-α to treat the astrocytes after OGD and detect the cell viability. We qualitatively found both TNF-α and p-P65 expression was inhibited through Western blot (Figure 8G). The MTT assay revealed that neutralization of TNF-α can also increase the astrocytes viability after OGD, just like Gap19 (*P* < 0.05, Figure 8H). This is a very interesting result, which further confirmed that Gap19 could alleviate the inflammatory response by inhibiting the opening of Cx43 hemichannels.

### Gap19 Attenuated LPS-Induced TLR4 Activation and Inflammation *in vitro*

**Figure 9:**
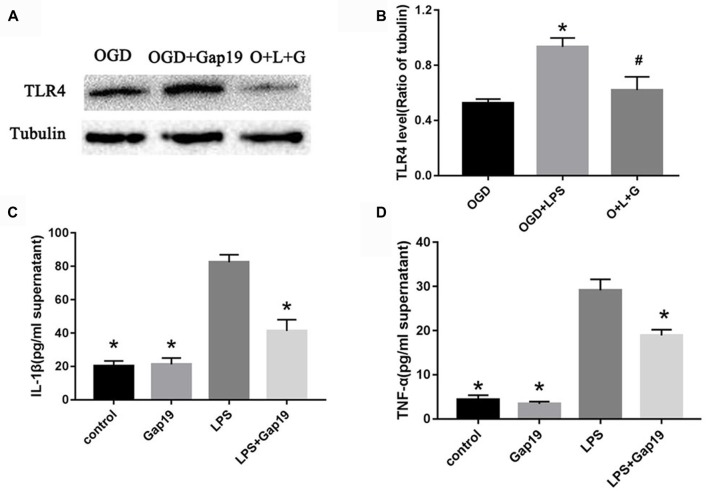
Gap19 attenuated LPS-induced TLR4 activation and inflammation *in vitro*. **(A)** Photomicrographs show TLR4 levels after LPS treatment using Western blot. The group O + L + G means OGD + LPS + Gap19 group. **(B)** Quantitative analysis confirmed that Gap19 treatment decreased TLR4 expression after LPS stimulation. **(C,D)** ELISA showed that Gap19 inhibited the release of IL-1β and TNF-α after LPS administration. Data represent the mean ± SEM (*n* = 3). *P < 0.05 vs. OGD group, ^#^*P* < 0.05 vs. OGD + LPS group.

Based on the above results, we hypothesized that Gap19 protected against cerebral I/R injury via downregulation of TLR4 signaling. LPS is one of the pathogen-associated molecular pattern that can specifically activate TLR4 signaling (Shao et al., 2013), and increase Cx43 hemichannels activity in astrocytes (Retamal et al., 2007). We conducted the LPS-induced experiment to further verify this hypothesis. The expression of TLR4 after LPS treatment was increased compared to the OGD group in the triple cell co-culture system (*P* < 0.05, Figures 9A,B). Gap19 treatment significantly attenuated TLR4 expression after LPS stimulation in OGD model. We further detected proinflammatory cytokine levels in the cell supernatant using ELISA. Gap19 treatment decreased the release of TNF-α and IL-1β in the culture medium after LPS stimulation (*P* < 0.05, Figures 9C,D). These results further corroborate that the protective effect of Gap19 occurred via downregulation of the TLR4 pathway.

## Discussion

The concept of the NVU was first proposed in 2003 by Lo and colleagues (Lo et al., 2003). Cerebrovascular disease research has gradually shifted focus from merely neurons or astrocytes to the NVU integral components, and it is considered a precondition for the screening of novel drugs and therapeutic target for stroke (Hu et al., 2017). Glial cells (astrocytes and oligodendrocytes) and BMECs are damaged via similar injury pathways, including glutamate toxicity, during ischemic injury (George and Steinberg, 2015). Ischemic damage also activates endogenous immune cells such as microglia (Kim et al., 2014). This study successfully established the cerebral I/R model *in* *vivo*/*vitro* and examined the role of Gap19 in cerebral I/R injury. Our results suggested that Gap19 reduced the infarct size and prevented the deterioration of the neurological function after cerebral ischemia and alleviated brain white matter damage. We further investigated the protection mechanism of Gap19. Result showed that Gap19 reduced the expression levels of Cx43, TLR4, MyD88 and NF-κB after ischemic injury and reduced the inflammatory response secondary to reperfusion.

We also investigated the mechanism about how Gap19 alleviating inflammation following stroke. We cultured primary neurons, astrocytes and cerebrovascular endothelial cells and constructed an NVU model for OGD treatment to simulate the *in vivo* process of cerebral I/R injury. Our result showed that when the concentration of Gap19 over 150 μM, the cell viability decreased compared with 100 μM. We think that high concentration strongly inhibits the Cx43 expression and decrease the gap junction formation, further interfering cell-to-cell communication. We detected the change of hemichannels activity on different treatment by EtBr uptake assay. Results showed that the opening of hemichannels increased after OGD and Gap19 could inhibit this effect on astrocytes. We also observed that Gap19 inhibited activation of the TLR4 pathway after OGD and inhibited the expression of pathway-related proteins, reduced the release of TNF-α and IL-1β, which was similar with the role of neutralization of TNF-α. Gap19 also decreased the expression of TLR4 in the LPS administration OGD model. Our results are the first to demonstrate that specific blocker of Cx43 hemichannels-Gap19 plays a vital role in protecting cerebral ischemia via inhibition of activation of the TLR4 pathways.

NVU consists of neurons, astrocytes and cerebrovascular endothelial cells, pericytes, basal membranes, and extracellular matrix (Stanimirovic and Friedman, 2012). Nerve cells are primarily connected with each other via gap junctions to communicate electrical signals, transmit chemical signals and metabolites to support the normal function of NVU cells (Giaume et al., 2010). Our study considered the NVU as the main research object and gap junctions as the main target. Cx43 is the main connexin protein that forms the gap junctions and hemichannels, and these proteins are expressed abundantly in brain, especially in astrocytes (D’hondt et al., 2014). Large amounts of hemichannels are opened after ischemia, causing ion disorders, loss of important metabolic substances, cell swelling and even death. Previous studies have shown that the use of gap junction inhibitors, such as glycyrrhetinic acid and its derivative carbenoxolone, improve blood-brain barrier permeability in mice, but the specificity is poor, and glycyrrhetinic acid interferes with other types of junctions (Takeuchi et al., 2011). Our previous studies demonstrated that the Cx43 mimetic peptides Gap26/Gap27 exhibited protective effects on the hypoxia/ischemia model in neonatal rats (Li et al., 2015). Hawat et al. (2010) found that Gap26 reduced infarct volume in myocardial ischemia reperfusion injury in rats. However, Gap26/27 also affected the formation of gap junctions when hemichannels are blocked.

Gap19 is a mimetic peptide that corresponds to a sequence in the CL of Cx43, which may disturb the interaction of C-terminal tail and CL of Cx43 (Abudara et al., 2014). The peptide may keep the gap junction open and hemichannel closed after ischemia with high specificity (Delmar et al., 2004). Wang N. et al. (2013) verified that Gap19 decreased the infarct volume after myocardial I/R in mice and alleviated cell oedema. Vicario et al. (2017) found that Gap19 specifically inhibited damage to human neuroblastoma cells after hypoxia/reoxygenation injury. Walrave et al. (2016) reported that TAT-Gap19 impaired hippocampal short-term spatial memory via inhibition of Cx43 hemichannels. Treatment with Gap19 decreased Cx43 expression in our study, which could further decrease the formation of Cx43 hemichannels. Our previous study found that Gap26 could decrease both total and dephosphorylated Cx43, and promote the internalization of Cx43 and the degradation of Cx43 in cytoplasm through the ubiquitin proteasome pathway (Li et al., 2015). In this study, Gap19 reduced infarction volume more than Gap26, exhibited better specificity and played a neuroprotective role in brain ischemia, and our results identified that Gap19 could significantly decrease the hemichannels activity on astrocytes but not on neurons and BMECs. Previous studies showed that Cx43 mainly expressed in astrocytes, rarely on neurons (Liu et al., 2012; Schulz et al., 2015), and could increase in BMECs after inflammation (Danesh-Meyer et al., 2012). In this NVU model, astrocytes were the critical one as bridge to connect the different components.

I/R is a pathological process that interrupts the blood flow of an organ, subsequent with reperfusion and reoxidation. Blood recirculation further aggravates tissue damage, which leads to a more severe inflammatory response, known as reperfusion injury (Eltzschig and Eckle, 2011). This injury occurs in a sterile environment and involves signaling events through pattern-recognition receptors (PRRs), such as TLRs, and the infiltration of blood immune cells of the innate and adaptive immune systems (Shichita et al., 2012). As we all know, microglia is the primary immune effector and can be activated by ischemia, releasing inflammatory cytokines (Morioka et al., 1993). And in brain, TLRs are constitutively expressed in microglia, but also in astrocyte and can be activated by endogenous damage-associated molecular patterns (DAMPs), such as ATP (Zhang et al., 2010), high mobility group box 1 protein (Yang et al., 2011), heat shock proteins, extracellular matrix proteins and so on. They are released from damaged brain cells after ischemia (Kim et al., 2014; Molteni et al., 2016). Increasing evidence suggests that TLR4 plays an important role in secondary brain injury following ischemia (Brea et al., 2011). A recent study demonstrated that TLR4^−/−^ mice decreased neuronal damage and apoptosis after global cerebral I/R (Hua et al., 2007). Hua et al. (2015) found that TLR4 blocker TAK-242 alleviated the inflammation after brain ischemia and played a neuroprotective role. Although TLR4 was primarily expressed in microglia in the central nervous system, these receptors are also expressed in astrocytes (Carpentier et al., 2008), neurons (Tang et al., 2007), and cerebrovascular endothelial cells (Grace et al., 2014) in some pathological environments. Our study demonstrated that Gap19 inhibited activation of the TLR4 signaling by MyD88-dependent pathway. Previous studies showed that treatment with TNF-α or IL-1β could increase membrane permeability through Cx43 hemichannels in astrocytes (Retamal et al., 2007) and upregulate the expression of Cx43 in microglia (Shaikh et al., 2012; Sáez et al., 2013). Our results revealed that Gap19 attenuated the expression of TNF-α and IL-1β after cerebral I/R injury which further suggests that the protective effect may occur via inhibition of the inflammatory response after reperfusion. Previous researches showed that after inflammation stimulation, microglia can also express Cx43 and communicate with each other through the gap junction (Eugenín et al., 2001; Shaikh et al., 2012; Sáez et al., 2013). In the future study, it will be valuable to add the microglia in this system for further study.

A recent study demonstrated that the Cx43 hemichannel may be a novel mediator of sterile inflammatory diseases (Li et al., 2018), which is consistent with the results of our study. This research further indicates that Cx43 is associated with inflammation. LPS is the main component of the cytoderm of Gram-negative bacteria and specifically activated the TLR4 pathway to induce an inflammatory response (Kawai and Akira, 2010). The *in vitro* model found that Gap19 reduced TLR4 and inflammatory cytokines expression after LPS stimulation. Previous studies reported that IL-1β and TNF-α released by activated microglia can inhibit the gap junction communication and increase the hemichannels activity on astrocyte (Même et al., 2006; Retamal et al., 2007). In other words, Gap19 may also decrease the opening of Cx43 hemichannels. All these data demonstrated that the inhibitory effect of Gap19 on the inflammation secondary to I/R injury may be associated with the inhibition of TLR4 signaling pathway. However, how the interaction of Gap19 and Cx43 causes inhibition of the TLR4 pathway remains unknown. We think that the mechanism may be similar to the treatment with Gap26. Previous study showed that the decrease of Cx43 expression after DNA hyper-methylation further activates the transcriptional activation of apoptosis suppressor genes (Yu S. C. et al., 2012). Li et al. (2016) found that hypoxia during pregnancy may lead to continuous whole-brain hypo-methylation in the fetal brain. Hence, does it affect the methylation of Cx43 DNA promoter region? This still requires further study.

## Conclusion

Our results indicate that Gap19 can decrease the brain infarct volume, prevent the deterioration of neurological deficit and alleviate white matter damage, and that Gap19 can also remarkably reduce the inflammatory response via inhibition of the TLR4 signaling pathway following *in vivo* MCAO and *in vitro* OGD. Therefore, Gap19 may be a potential therapeutic drug for cerebral ischemic injury which associated with NVU disruption.

However, whether Gap19 affects TRIF-mediated inflammatory responses, which is the other pathway of TLR4, is not known. Future experiments should address this relationship. We did not verify the inhibition of Gap19 on the TLR4 pathway at the gene expression level, and more studies are required to further validate these results.

## Author Contributions

YC, LW and HY: conceived and designed the experiments; contributed to the writing of the manuscript. LW, LZ and LY: performed the experiments. HY, YL and LW: analyzed the data. LW, XL and BC: contributed reagents, materials and analysis tools. All authors approved the final manuscript.

## Conflict of Interest Statement

The authors declare that the research was conducted in the absence of any commercial or financial relationships that could be construed as a potential conflict of interest.
